# Fe(II)Cl_2_ amendment suppresses pond methane emissions by stimulating iron-dependent anaerobic oxidation of methane

**DOI:** 10.1093/femsec/fiae061

**Published:** 2024-04-17

**Authors:** Quinten Struik, José R Paranaíba, Martyna Glodowska, Sarian Kosten, Berber M J W Meulepas, Ana B Rios-Miguel, Mike S M Jetten, Miquel Lürling, Guido Waajen, Thomas P A Nijman, Annelies J Veraart

**Affiliations:** Department of Ecology, Radboud Institute for Biological and Environmental Sciences, Radboud University, 6525 AJ, Nijmegen, The Netherlands; Department of Ecology, Radboud Institute for Biological and Environmental Sciences, Radboud University, 6525 AJ, Nijmegen, The Netherlands; Department of Microbiology, Radboud Institute for Biological and Environmental Sciences, Radboud University, 6525 AJ, Nijmegen, The Netherlands; Department of Ecology, Radboud Institute for Biological and Environmental Sciences, Radboud University, 6525 AJ, Nijmegen, The Netherlands; Department of Ecology, Radboud Institute for Biological and Environmental Sciences, Radboud University, 6525 AJ, Nijmegen, The Netherlands; Department of Microbiology, Radboud Institute for Biological and Environmental Sciences, Radboud University, 6525 AJ, Nijmegen, The Netherlands; Department of Microbiology, Radboud Institute for Biological and Environmental Sciences, Radboud University, 6525 AJ, Nijmegen, The Netherlands; Aquatic Ecology & Water Quality Management Group, Department of Environmental Sciences, Wageningen University, PO Box 47, 6700 AA, Wageningen, The Netherlands; Water Authority Brabantse Delta, 4836 AA, Breda, The Netherlands; Department of Ecology, Radboud Institute for Biological and Environmental Sciences, Radboud University, 6525 AJ, Nijmegen, The Netherlands; Department of Ecology, Radboud Institute for Biological and Environmental Sciences, Radboud University, 6525 AJ, Nijmegen, The Netherlands

**Keywords:** bioremediation, Fe-AOM, freshwater sediment, geoengineering, Methanogenesis, mitigation

## Abstract

Aquatic ecosystems are large contributors to global methane (CH_4_) emissions. Eutrophication significantly enhances CH_4_-production as it stimulates methanogenesis. Mitigation measures aimed at reducing eutrophication, such as the addition of metal salts to immobilize phosphate (PO_4_^3−^), are now common practice. However, the effects of such remedies on methanogenic and methanotrophic communities—and therefore on CH_4_-cycling—remain largely unexplored. Here, we demonstrate that Fe(II)Cl_2_ addition, used as PO_4_^3-^ binder, differentially affected microbial CH_4_ cycling-processes in field experiments and batch incubations. In the field experiments, carried out in enclosures in a eutrophic pond, Fe(II)Cl_2_ application lowered *in-situ* CH_4_ emissions by lowering net CH_4_-production, while sediment aerobic CH_4_-oxidation rates—as found in batch incubations of sediment from the enclosures—did not differ from control. In Fe(II)Cl_2_-treated sediments, a decrease in net CH_4_-production rates could be attributed to the stimulation of iron-dependent anaerobic CH_4_-oxidation (Fe-AOM). In batch incubations, anaerobic CH_4_-oxidation and Fe(II)-production started immediately after CH_4_ addition, indicating Fe-AOM, likely enabled by favorable indigenous iron cycling conditions and the present methanotroph community in the pond sediment. 16S rRNA sequencing data confirmed the presence of anaerobic CH_4_-oxidizing archaea and both iron-reducing and iron-oxidizing bacteria in the tested sediments. Thus, besides combatting eutrophication, Fe(II)Cl_2_ application can mitigate CH_4_ emissions by reducing microbial net CH_4_-production and stimulating Fe-AOM.

## Teaser

Fe(II)Cl2 application combat eutrophication and reduce CH4 emissions through increased Fe-dependent anaerobic CH4-oxidation.

## Introduction

Aquatic ecosystems are responsible for half of the global CH_4_ emissions (Rosentreter et al. 2021). CH_4_ release from organic sediments is strongly driven by eutrophication (Davidson et al. 2018), which remains a significant and ongoing worldwide problem (Beaulieu et al. 2019). Eutrophication increases autochthonous organic matter production, which precludes substrate limitation and promotes methanogenesis, and hence is estimated to increase CH_4_ emissions by 30%–90%, making eutrophic waters hotspots for CH_4_ emission (Attermeyer et al. 2016, Beaulieu et al. 2019). Besides nutrient-load reduction, geo-engineering techniques are increasingly used to combat eutrophication. These include techniques using PO_4_^3−^ (P)-binding compounds such as Fe(II)Cl_2_, lanthanum-modified bentonite clay, and aluminum-modified zeolite (Jančula and Maršálek 2011). However, there is little insight into how eutrophication remediation strategies affect aerobic and anaerobic CH_4_-cycling microorganisms, and how these affect CH_4_ emissions (Jančula and Maršálek 2011, Nijman et al. 2022).

Therefore, in this study, we tested the effect of the P-binding agent Fe(II)Cl_2_ on microbial CH_4_-cycling in field experiments and batch incubations. In oxic layers of Fe-rich sediments the majority of the phosphorus (P) is bound to ferric iron (Fe(III)) (Parsons et al. 2017). Once the bound Fe(III)-P reaches the anoxic zone of the sediment, bound P is released from the Fe(III)-P complex, as a consequence of the reduction and accompanying dissolution of the Fe particles (Cooke et al. 1993). Subsequently, in the anoxic zone of aquatic sediments, in the presence of Fe(II), P is known to form vivianite (Fe(II)_3_(PO_4_)_2_•8 H_2_O) through authigenesis. Vivianite is a hydrated ferrous phosphate mineral that immobilizes phosphate (Walpersdorf et al. 2013, Rothe et al. 2014, Liu et al. 2018, Heinrich et al. 2021). However, when exposed to alternative electron acceptors (e.g. O_2_, NO_3_^−^, NO_2_, SO_4_^2−^) up to 50% of the Fe(II) present in vivianite can be oxidized to poorly crystalline mixed-valence or ferric Fe(III)-P molecules, increasing the bioavailable Fe(III) in the sediment (Nielsen and Nielsen 1998, Miot et al. 2009, Kusunoki et al. 2015, Rothe et al. 2016). Additionally, ferrous Fe-salts like Fe(II)Cl_2_ that are directly applied in the sediments can be directly oxidized with O_2_ or NO_3_^−^ (Benz et al. 1998, Nielsen and Nielsen 1998, Oikonomidis et al. 2010). Fe(II)Cl_2_ application could therefore, through direct Fe(II)Cl_2_ oxidation or through vivianite oxidation, substantially increase the bioavailable Fe(III) concentrations in the sediment.

CH_4_ formation (methanogenesis) is a microbial process commonly taking place in anoxic sediments (Segers 1998). CH_4_ can be oxidized to CO_2_ by methanotrophic microorganisms, either aerobically, using O_2_ as electron acceptor, or anaerobically, using different alternative electron acceptors (e.g. NO_3_^−^, Fe(III), Mn^4+^ or SO_4_^2−^) depending on their availability (Segers 1998). Anaerobic oxidation of CH_4_ (AOM) can oxidize up to 50–90% of the CH_4_ formed in natural aquatic sediments and is suggested to be among the main processes involved in lowering the natural aquatic CH_4_ emissions (Norði et al. 2013, Segarra et al. 2015, Weber et al. 2016, Vigderovich et al. 2022). In Fe(III) rich freshwater sediments, Fe-dependent anaerobic CH_4_-oxidation (Fe-AOM) (Equation 1), has been found to oxidize up to 15% of the produced CH_4_ (Sivan et al. 2011, Ettwig et al. 2016, Scheller et al. 2016), by exploiting soluble and nanophase Fe(III) as electron acceptors (Norði et al. 2013).


(1) \begin{eqnarray*}
{\mathrm{C}}{{\mathrm{H}}}_4 + {\mathrm{\ }}2{{\mathrm{H}}}_2{\mathrm{O}} + 8{\mathrm{F}}{{\mathrm{e}}}^{3 + } \to {\mathrm{\ C}}{{\mathrm{O}}}_2 + 8{{\mathrm{H}}}^ + + 8{\mathrm{F}}{{\mathrm{e}}}^{2 + }
\end{eqnarray*}

Fe-AOM is mediated by anaerobic CH_4_-oxidizing archaea (ANME), which belong to three distinct clades (ANME 1–3) (Weber et al. 2017). Members of the ANME-1 clade are highly diverse, and fall in the order of *Methanophagales*. ANME-2 is comprised of three families, all in the order of *Methanosarcinales*, of which one is observed in freshwaters; the *Methanoperedenaceae* (ANME-2d), and two are observed in marine systems: *Methanocomedenaceae* and *Methanogasteraceae*. ANME-3 is closely related to known methanogens, and represents a novel genus within the family of *Methanosarcinaceae* (Chadwick et al. 2022).

Since Fe(II)Cl_2_ addition potentially leads to elevated Fe(III) concentrations in the sediment, it is hypothesized that this could lead to a significant change in the contribution of Fe-AOM to the CH_4_ cycle in the long term, lowering both internal PO_4_^3−^ concentrations and *in situ* CH_4_ emissions.

Here we aim to unravel the effects of Fe(II)Cl_2_ addition on the aquatic carbon cycle. With this study we generate new insights into how bioremediation techniques -besides combating eutrophication- can help in reducing aquatic CH_4_ emissions, contributing to climate-smart water management.

## Methodology

### Field description

To evaluate the effect of Fe(II)Cl_2_ on CH_4_ dynamics, an in-situ enclosure experiment was established inside an eutrophic pond. The pond is located at the property of Water Authority ‘Brabantse Delta’, Breda, The Netherlands (51°33′45.5″N 4°46′58.7″E). The pond consists of an open water system, which is connected to a surrounding channel system and encompasses a water surface area of roughly 14 500 m^2^, with an average water depth of 1.1 m (Kang et al. 2023). On average, the total water N and P concentrations range from 0.46 to 4 mg N L^−1^ and 0.03–0.22 mg P L^−1^ and the bioavailable P fraction (top 6 cm sediment) was on average 0.91 mg g dw^-1^. More details on site description can be found in Kang et al. (2023).

### Experimental setup

In March 2020, a total of 8 transparent Perspex cylindrical enclosures (1.05 m diameter, 1.30 m height and a total volume of 865 L, open at the top and bottom) were firmly pushed into the ponds’ sediment (approximately 0.2 m deep) (Kang et al. 2023). The enclosures were positioned in line, at a 30 cm interval, and the top-edge of the enclosures extended approximately 25 cm above the water surface. After placement (9^th^ March 2020), the enclosures were left to settle for approximately 1.5 months. On the 8^th^ of April 2020 the enclosures were randomly treated with Fe(II)Cl_2_ (N=4) or left as a control (N=4) (Kang et al. 2023). While the control enclosures were left undisturbed, the Fe(II)Cl_2_ treatment received 19.1 g of crystalline Fe(II)Cl_2_ (CAS-Nr.: 13478–10–9, Honeywell) that was dissolved in 200 ml anoxic acetate buffer, to obtain a 0.48 M Fe(II)Cl_2_-solution (pH = 4.2). In total 20 ml of this solution was directly injected into the upper 6 cm of the sediment at 10 different spots, which were randomly chosen from a 16-squared grid placed on top of each enclosure (Kang et al. 2023). The applied dosage of Fe(II)Cl_2_ was based on the amount of bioavailable P in the first 6 cm of the sediment, to target a 1.5 molar ratio (Fe: P), and is further described in Kang et al. (2023). In the Fe(II)-treated sediments, Fe(III) accounted for between 66% and 91% of the total Fe, in the control it was 81% (Kang et al. 2023).

### Field measurements

To quantify the effect of the Fe(II)Cl_2_ treatment on the CH_4_ emissions from the pond enclosures, we measured CH_4_ emissions (totaling 5 sampling occasions) from the 15^th^ of September 2021 (17 months after Fe(II)Cl_2_ addition) until the 10^th^ of November 2021. We measured CH_4_ emission after over a year of Fe(II)Cl_2_ application because in general differences in long-term ecosystem effects become apparent after a complete growing season, due to indirect effects of P-binding agents on the pond-community, for example by changing macrophyte development (Nijman et al. 2022). During the measuring period, O_2_ concentrations 1 cm above the sediment-water interface ranged between 0.2–6.3 mg L^−1^ for control enclosures, and between 0.1 and 8.2 mg L^−1^ in Fe(II)Cl_2_-treated enclosures. Additionally, the diffusive CH_4_ emissions from the enclosures were measured biweekly. To limit potential CH_4_-emission variation caused by measurement time, all measurements were performed around midday, between 11:00 and 14:00 h, hence we did not account for possible diel variability. Fluxes were measured using the floating chamber method, and ebullition was measured by permanently installed bubble traps (Almeida et al. 2016). The bubble traps consisted of up-side down funnels (surface area of 0.033 m^2^) connected to glass (1000 ml) collection bottles filled with pond water as exemplified in Almeida et al. (2016). During the study duration no bubbles were caught, indicating ebullition was of minor importance. The diffusive flux was measured using a transparent closed floating chamber (height 30 cm, diameter 28.8 cm) that was placed on top of the water column of the enclosure. The chamber was connected to an Ultra-portable greenhouse gas analyzer (UGGA—Los Gatos ®) that was used to quantify the change in CH_4_ concentration over a period of 3 min, using a closed loop system (Almeida et al. 2016). After placing the chamber, we waited for approximately 1 min for the flux to become stable, to ensure we were not underestimating or overestimating the CH_4_ flux. The UGGA has an operating range of 0.01–100 ppm for CH_4_, with a 10-second response time, and a measuring frequency of 1 Hz. Although wind speed is a known driver of water-air fluxes of CH_4_, due to its effect on turbulent mixing, wind effects were limited because of the enclosure-edges prevented wind-driven wave action. The flux was calculated using equation 2.


(2) \begin{eqnarray*}
F = \frac{V}{A}*{\mathrm{slope}}*\ \frac{{P*F1*F2}}{{R*T}}
\end{eqnarray*}

Where *F* is the gas flux (mg m^−2^ d^−1^), *V* is chamber volume (L), *A* is the area of the chamber surface (m^2^), ‘*slope*’ is the CH_4_ concentration change over time (ppm s^−1^); *P* is the atmospheric pressure (atm), *T* is the temperature in (^o^K); *R* is the gas constant = 8.205746 × 10^−5^ m^3^ atm mol^−1^ K^−1^; *F*1 is the molecular weight of CH_4_ (16 g mol^−1^); *F*2 is the conversion from seconds to days.

At the end of the experiment, 3 sediment cores (diameter 6 cm, height 60 cm) were collected in each enclosure using a UWITEC sediment corer (UWITEC, Mondsee, Austria). The sediment of one core was sliced every 1 cm and analyzed for total P and total Fe. The other cores were used for batch incubations.

### Incubation experiments

**Figure 1.**
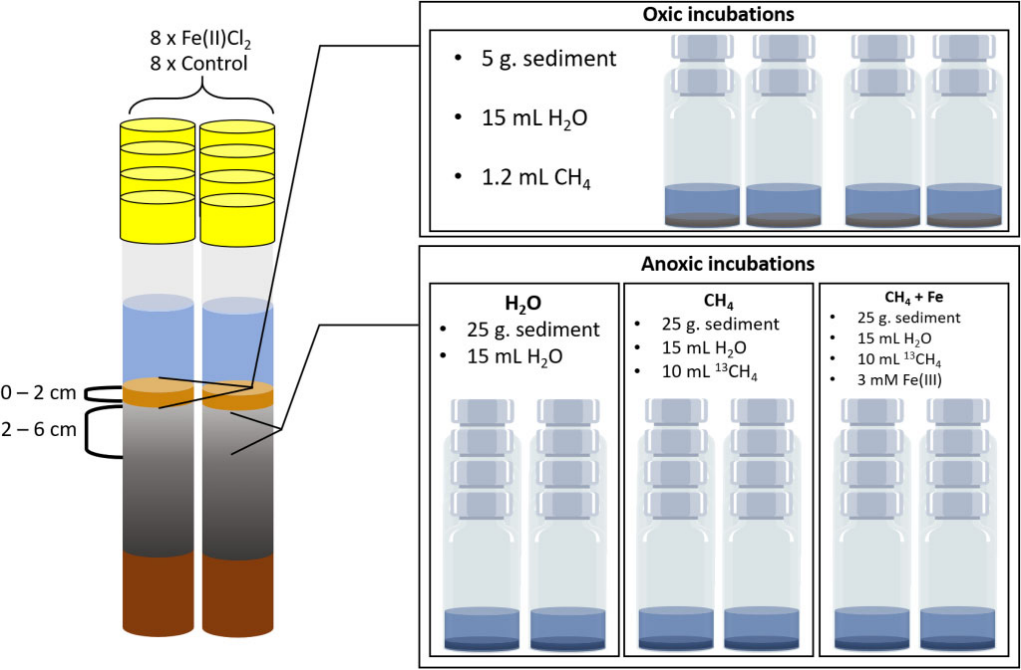
Schematic overview of the sampling design. Where 8 sediment cores originating from the enclosures were sliced for either oxic incubations or anoxic incubations, following different treatments. Source: Partly created using Canva by the authors.

Four sets of incubation experiments were performed (Fig. 1). To evaluate the effect of the Fe(II)Cl_2_ treatment in the enclosures, enclosure sediments were incubated under oxic and anoxic conditions allowing for rate measurements of net CH_4_-production and CH_4_-oxidation. First, the aerobic top layer of the sediment cores (first 2 cm), as observed by a change in sediment color from light (oxidized) to black (anoxic), was used to quantify the aerobic CH_4_-oxidation rate, and subsequently to determine sediment moisture content by drying the sediments for 3 days at 70°C. Next, another intact sediment core was transferred to an anaerobic chamber (<10 ppm O_2_), where the top 2 cm was removed. The layer between 2 and 6 cm sediment-depth was used to quantify sediment CH_4_-production and potential Fe-dependent anaerobic CH_4_-oxidation (incubations 2, 3 and 4).

To measure potential aerobic CH_4_-oxidation rates, 5 g of sediment + 10 ml of oxygenated (∼7 mg L^−1^) enclosure water was incubated under atmospheric (oxic) conditions in 120 ml gastight serum bottles. After sediment transfer, the bottles were capped and injected with 1.2 ml (= 1%) 99% CH_4_ gas, as in Nijman et al. (2022). The sediments were incubated at 18°C and mixed continuously using a gyratory shaker (105 r/m). The CH_4_ concentration in the headspace was measured directly after injection of 1.2 ml (=1%) CH_4_, and daily at 10:00, 13:00, 17:00 h during 4 days, using a gas chromatograph equipped with flame ionization detection (Hewlett Packard HP 5890 Series II Gas Chromatograph, Agilent Technologies, California, USA). To ensure the incubation bottles did not become anoxic, we measured O_2_ concentrations in all the bottles using a Microx TX3 oxygen microsensor (flat-broken needle tip, Presens, Germany), at the end of the incubation experiments.

To determine net CH_4_-production rates (= CH_4_-production—anaerobic CH_4_-oxidation), direct anaerobic CH_4_-oxidation rates and potential Fe-AOM (all corrected for 82% sediment moisture content), we incubated 25 g of sediment (2–6 cm depth) + 15 ml anoxic enclosure water in 140 ml gastight serum bottles under fully anoxic conditions. Anoxia was confirmed using Microx TX3 oxygen microsensors (flat-broken needle tip, Presens, Germany) in all bottles. For these anoxic incubations, different treatments were used: 1) ‘H_2_O’– containing only sediment and water, used to determine the net CH_4_-production under control conditions; 2) ‘CH_4_’– supplemented with 10 ml of 99% ^13^CH_4_; and 3) ‘CH_4_ + Fe’—supplemented with 10 ml of 99% ^13^CH_4_ and 3 mM ferrihydrite (Fe(III)_2_O_3_·0.5H_2_O) to determine potential rates of Fe-AOM. The bottles were kept in a climate-controlled room at 18°C in the dark. Throughout 129 days, approximately once per month the total CH_4_, ^13^CO_2_ and ^12^CO_2_ concentrations of the headspace were determined repeatedly by gas chromatography coupled to mass spectrometry (Trace DSQ II, Thermo Finnigan, Austin TX, USA). The concentration of CH_4_ was quantified as described for aerobic CH_4_-oxidation. Due to simultaneous CH_4_-production and CH_4_-consumption in anoxic incubations, the AOM potential was monitored by the increase in ^13/12^CO_2_ rather than the change in CH_4_ concentration. To test for the potential of Fe-AOM in these incubations, we simultaneously measured the dissolved Fe(II), total Fe(II) and total Fe in water and sediment using a colorimetric ferrozine assay (Schaedler et al. 2018).

### 16S rRNA sequencing

To explore the microbial potential for Fe-AOM we extracted DNA from the original *in situ* enclosure sediment which was used for the anaerobic incubations (2–6 cm) and from the anoxic batch incubations at the end of the 130-day incubation period. DNA was extracted using the PowerSoil DNA extraction kit (DNeasy PowerSoil Pro Kit, QIAGEN, Hilden, Germany), according to the manufacturer's protocol. The concentration of DNA was quantified using Qubit® 2.0 Fluorometer with DNA HS kits (Life Technologies, Carlsbad, CA, USA). 16S rRNA gene amplicon sequencing was performed by Macrogen (Amsterdam, The Netherlands) using the Illumina MiSeq Next Generation Sequencing platform*. Paired-end libraries were constructed using the Illumina Herculase II Fusion DNA Polymerase Nextera XT Index Kit V2 (Illumina, Eindhoven, Netherlands). The primers used for bacterial amplification were Bac341F (5′-CCTACGGGNGGCWGCAG-3′; (Herlemann et al. 2011) and Bac806R (5′-GGACTACHVGGGTWTCTAAT-3′; (Caporaso et al. 2012). Archaeal amplification was performed with primers Arch349F (5′-GYGCASCAGKCGMGAAW-3′) and Arch806R (5′-GGACTACVSGGGTATCTAAT-3′; (Takai and Horikoshi 2000). Analysis of the 16S rRNA sequencing output files was performed within R version 3.5.1 (Team 2013) using the DADA2 pipeline (Callahan et al. 2016). After checking the reads quality of the samples, the length of the reads were trimmed using the following parameter: truncLen=c(280 200). Taxonomic assignment of the reads was up to the species level when possible, using the Silva non-redundant database version 132 (Yilmaz et al. 2014). Count data were normalized to relative abundances. Further data analysis and the creation of amplicon sequence variant (ASV) tables at different taxonomical levels were performed using the *phyloseq* and *microbiome* packages (McMurdie and Holmes 2013).

*All raw sequencing data have been deposited at the read sequence archive (SRA) database of the NCBI under the BioProject ID PRJNA966799.

### Statistical approach

All statistical tests were executed in R, developed by the R Core Team (2013). We tested the data for normal distribution and assumptions for every test executed. To test for differences in CH_4_ emissions between treatments in the field, a linear model (LM) was used on the log-transformed data (CH_4_ emissions being the dependent variable, and treatments + date the independent variables). Additionally, to test for differences between dissolved Fe(II) concentrations and changes in ratio ^13/12^CO_2_ between treatments, an analysis of variance (two-way ANOVA) combined with Tukey's post-hoc test was used. Furthermore, the difference in net CH_4_-production rates and aerobic CH_4_-oxidation rates between treatments was tested using a Welch's T-test for unequal variance and a Student's T-test, respectively. To visualize and test linear relationships for the increase in ^13/12^CO_2_ we used a LM (^13/12^CO_2_ being the dependent variable, and days of incubation * treatment * treatment the independent variables). Potential CH_4_-oxidation and CH_4_-production rates were calculated using the slope of the linear part of the potential CH_4_-oxidation curve divided by the absolute dry weight (g) of the incubated sediment, and corrected for time. Only the linear part of the slope was used to minimize the effects of substrate limitation and effects of (by)product inhibition on the calculation of potential CH_4_-oxidation rates.

## Results and discussion

Fe(II)Cl_2_ addition effectively lowered PO_4_^3-^ concentrations in the enclosures, lowering the PO_4_^3-^ concentration from 2.2 ± 0.4 to 1 ± 0.4 µM in the surface water, and considerably boosted Fe concentrations in sediment and water (Table 1). This is in line with previous findings, where under favorable Fe: P ratios and redox conditions Fe addition successfully locked P into lake sediments (Wang and Jiang 2016). Here, in Fe(II)Cl_2_-treated enclosures, up to 91% of the Fe was present in the form of Fe(III) (Kang et al. 2023).

**Table 1.** Average nitrate and trace metal concentrations in surface water and the sediment top layer (5 cm) for control and Fe(II)Cl_2_-treated enclosures at the end of the enclosure experiment (Nov 2021).

	Water					Sediment	
Enclosure	PO_4_^3−^ µM	NO_3_^−^ µM	Mn µM	Total Fe µM	SO_4_^2−^ µM	Mn mM kg^−1^ DW	Total Fe mM kg^−1^ DW
Control	2.2±0.42	0.3±0.25	8±1.3	61±15	14.5±6	5.1±2.3	180±52
Fe(II)Cl_2_	1±0.38	0.1±0.05	10±5.6	280±153	21±17	2.4±1.7	207±137

**Figure 2.**
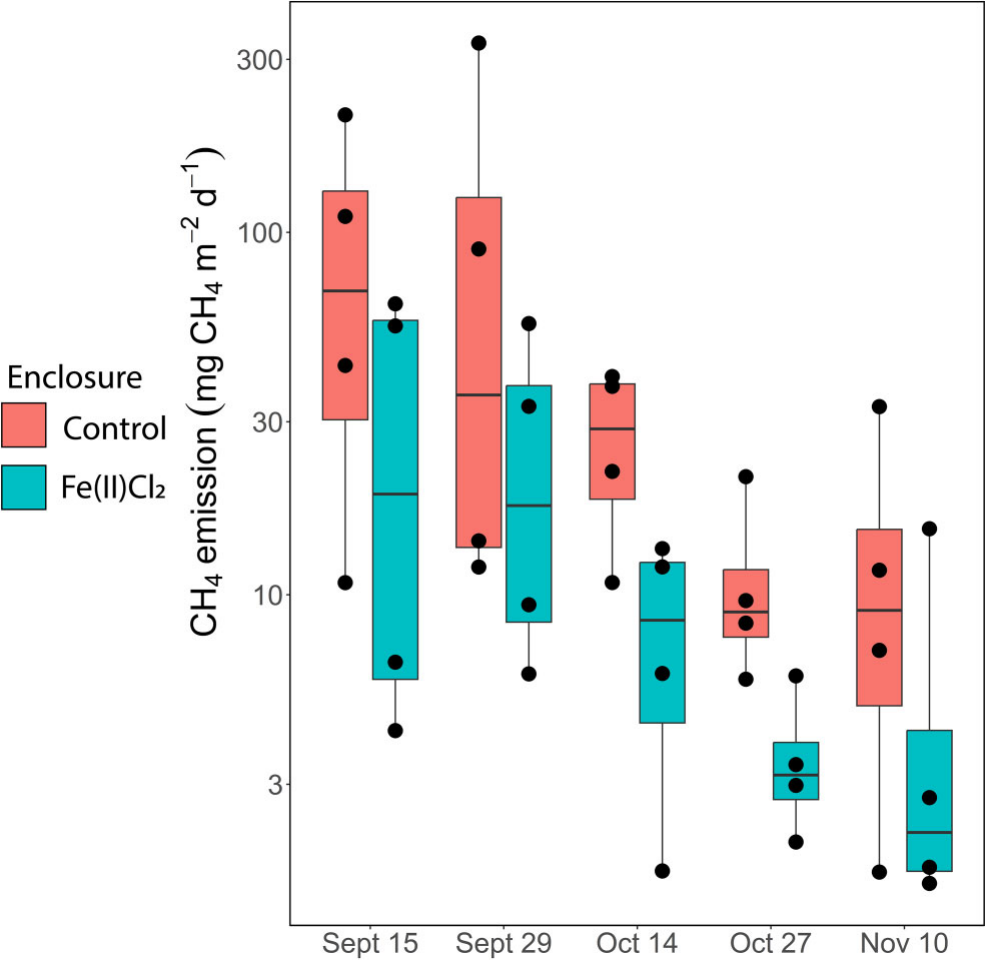
Water-atmosphere CH_4_ emissions from the enclosures in the field Y-axis is on log10 scale. Boxes show the median and first and third quartiles, whiskers indicate upper and lower quartiles, and dots indicate individual sampling points. CH_4_ emissions from the Fe(II)Cl_2_-treated enclosures (N=4) were significantly lower compared to controls (N=4) based on a linear model (LM) (*P*<0.001, r^2^=0.43, df=34, F=6.9).

CH_4_ emissions of the Fe(II)Cl_2_-treated enclosures were on average 3.5 times lower than those of unamended (*P*<0.001, r^2^=0.43, df=34, F=6.9, LM; Fig. 2), where control CH_4_ emissions averaged 52 ± 78 mg CH_4_ m^−2^ d^−1^ and Fe(II)Cl_2_-treated enclosures 15 ±14 CH_4_ m^−2^ d^−1^, suggesting that Fe(II)Cl_2_ addition lowered CH_4_ emissions. Phosphorus control has previously been found to lower CH_4_ emissions through indirect effects of oligotrophication on the aquatic foodweb (Nijman et al. 2022). Additionally, we observed a general descending CH_4_ emission intensity over time (Fig. 2), which is likely due to seasonality, where in colder months CH_4_ emissions decreases. Here, we test if the effects on CH_4_ emission can also result from direct effects on microbial CH_4_-production and oxidation in the sediment.

**Figure 3.**
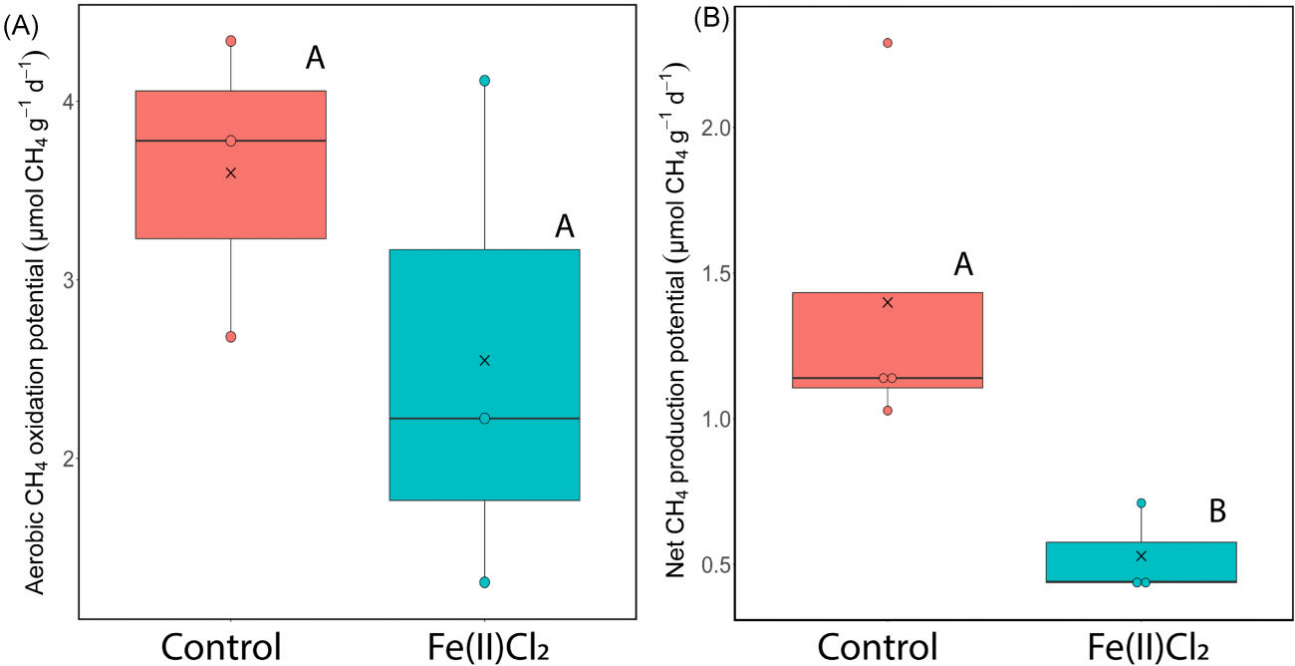
Batch incubation of sediments from the Fe(II)Cl_2_-treated enclosures (blue) and the control enclosures (red). (A) Aerobic CH_4_-oxidation potential (calculated from the linear part of the trend) per g. dry weight sediment per day (µmol CH_4_ g^−1^ d^−1^). No significant difference was observed between treatments (T-test, *P*=0.34, df=5, t=1.1). Boxes show the median and first and third quartiles, whiskers indicate upper and lower quartiles, dots indicate individual sampling points, and ‘x’ indicates the mean. (B) The net potential CH_4_-production rate of the sediment originating from different enclosures, expressed as µmol CH_4_ per g. dry weight sediment per day (µmol CH_4_ g^−1^ d^−1^). Sediments from control enclosures have a marginally significant higher net CH_4_-production rate, compared to the sediments from the Fe(II)Cl_2_-treatment (T-test, *P*=0.05, df=3.5, t=2.8).

The aerobic CH_4_-oxidation rates of Fe(II)Cl_2_-treated sediments (average: 2.4 ± 1.2 µmol CH_4_ g^−1^ d^−1^) were not significantly different than rates observed in the control-enclosure sediments (average: 3.6 ± 0.7 µmol CH_4_ g^−1^ d^−1^; *P*=0.34, df=5, t=1.1; T-test) (Fig. 3A), implying that aerobic CH_4_-oxidation played a minor role in lowering CH_4_ emissions from the Fe(II)Cl_2_-treated enclosures. However, sediments from the Fe(II)Cl_2_-treated enclosures showed ∼3 times lower net CH_4_-production rate compared to the control sediments (Fe(II)Cl_2_ average: 0.53 ± 0.13 µmol CH_4_ g^−1^ d^−1^, control average: 1.4 ± 0.52 µmol CH_4_ g^−1^ d^−1^, *P*=0.05, df=3.5 t=2.8, T-test, Fig. 3B and S1A). Hence, given that: (i) sediment and water samples from Fe(II)Cl_2_-treated enclosures showed enrichment in Fe(III) content, (ii) other potential electron acceptors were only found in low concentrations (Table 1, and in Kang et al. 2023), and (iii) potential aerobic CH_4_-oxidation rates were comparable between control and Fe(II)Cl_2_-treated enclosures, while in contrast net CH_4_-production rates—including potential anaerobic methane oxidation—were different, we hypothesized that Fe-AOM played an important role in lowering the *in situ* CH_4_ emissions.

**Figure 4.**
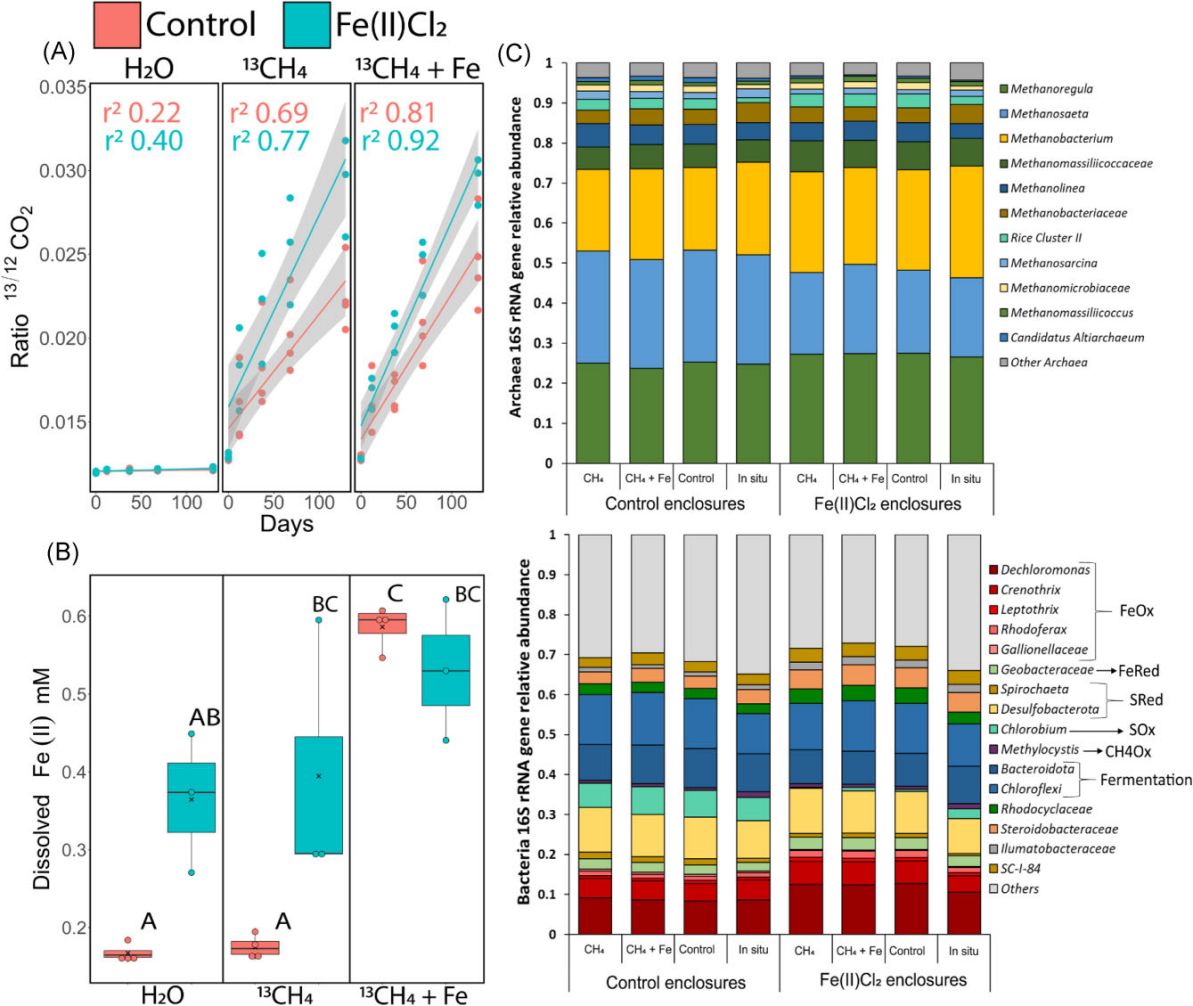
Batch incubation of sediments from the Fe(II)Cl_2_-treated enclosures (blue) and the control enclosures (red). (A) Increase in ^13/12^CO_2_, with a significant difference between Fe(II)Cl_2_-treated sediments and control based on a LM (*P*<0.0001, r^2^=0.88, df=90, F=69.5). Shaded areas show the 95% confidence interval. (B) Concentrations of dissolved Fe(II) (mM) at the end of the experiment (day 129). Boxes show the median and first and third quartiles, whiskers indicate upper and lower quartiles, dots indicate individual sampling points, and X indicates the mean. Letters depict which groups significantly differ from each other based on a two-way ANOVA (*P<*0.01, df=2, F=6.3) combined with a Tukey post-hoc test (Table S2) (C) 16S rRNA gene sequencing data, “in situ” refers to the microbial community originating from the original enclosure sediment, whereas the other three samples are taken from the incubations at the end of the experiment from each serum bottle (day 130).

We monitored AOM rates and Fe(III) reduction in incubations of enclosure sediments with and without Fe(III) addition. We found clear indications that the AOM-potential was higher in sediments originating from the Fe(II)Cl_2_-treated enclosures than in sediments originating from untreated controls, as seen by their significantly higher ^13/12^CO_2_ ratio after incubation with ^13^CH_4_ (average ^13/12^CO_2_ ratio at the end of the incubation period: control 0.22 ± 0.001, Fe(II)Cl_2_ 0.29 ± 0.002) (Fig. 4A and S1B, Table S1). Moreover, the simultaneous addition of ^13^CH_4_ and Fe(III) to control sediments led to higher AOM-rates compared to the ^13^CH_4_-only addition, and also significantly boosted Fe(II)-production, suggesting that Fe-AOM is a key pathway (Fig. 4B and S1C). This finding is in line with a study on anaerobic methanotroph bioreactors inoculated with paddy soil, where ferrihydrite and ^13^CH_4_ addition also resulted in a rapid onset of ^13^CO_2_ production, indicating the occurrence of Fe-AOM (He et al. 2021).

Microbial community analysis revealed the presence of Fe-cycling and CH_4_-cycling microorganisms in the control and Fe(II)Cl_2_-treated sediments, implying there is Fe-AOM-potential (Fig. 4C). The presence of Fe(II)-oxidizing bacteria supports the hypothesis that Fe(II) originating from Fe(II)Cl_2_ can be oxidized to Fe(III), providing an electron acceptor for Fe-AOM (Benz et al. 1998, Nielsen and Nielsen 1998, Oikonomidis et al. 2010). The archaeal community mainly consisted of methanogens, explaining the observed high CH_4_ emissions. Known CH-producing taxa such as *Methanosarcinales* and *Methanomicrobia* also include members capable of CH_4_-oxidation via reverse methanogenesis, possibly involved in Fe-AOM (Oni and Friedrich 2017).

*Methanoperedens* species known to mediate both nitrate-dependent AOM and Fe-AOM (Legierse et al. 2023) were also present, albeit at low abundance (below 0.06% in both treatments). Additionally, in both control and Fe(II)Cl_2_-supplemented enclosure sediments, the methanogen *Methanomassiliicoccaceae*, was abundant (control 5%, Fe(II)Cl_2_ 7%), suggesting its involvement in Fe-AOM (He et al. 2021). Although the Fe(II)Cl_2_-treated sediments had a slightly higher abundance of Fe-cycling bacteria, the control sediments already contained Fe-oxidizers and Fe-reducers, indicating the functional capacity for Fe-AOM within the indigenous microbial community. This also explains the rapid—compared to Weber et al. (2017)—onset of Fe-AOM in our incubations. Furthermore, the relative abundance of sulphide-oxidizing phototrophic bacteria such as *Chlorobium* was substantially lower in the Fe(II)Cl_2_-treated sediments (2.5%, compared to the control 5.7%) which was likely related to the lower availability of sulphides that may have precipitated in the form of iron sulphide minerals.

**Figure 5.**
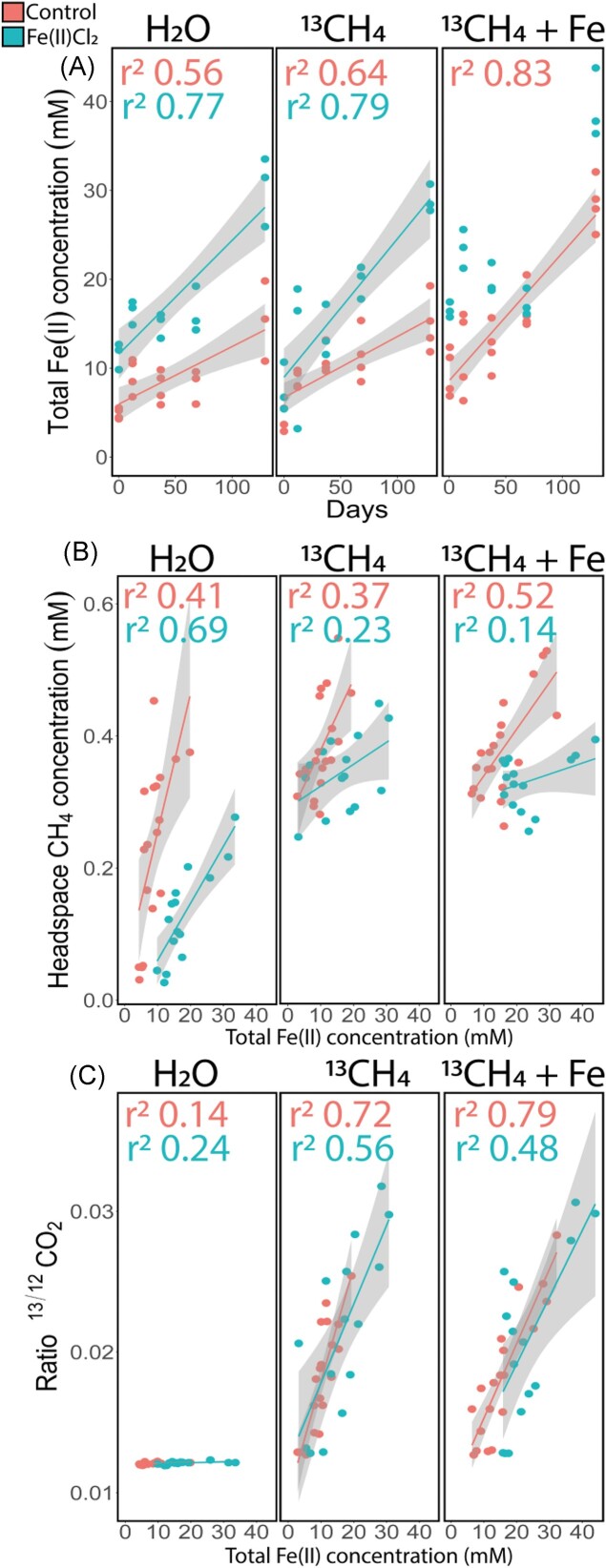
Batch incubation of sediments from the Fe(II)Cl_2_-treated enclosures (blue) and the control enclosures (red). Shaded areas show the 95% confidence interval and the dots indicate individual sampling points (A) Increase in total Fe(II) concentrations (mM) throughout different times during the incubation experiment. The Fe(II)Cl_2_ treated sediments had significantly higher total Fe(II) concentrations (mM) over time (LM, *P*=0.003, r^2^=0.80, df=91, F=37.7). (B) The headspace CH_4_ concentrations correlated to total Fe(II) concentrations (mM) (*P*=<0.0001). Total headspace CH_4_ concentration were lower in Fe(II)Cl_2_-treated sediments (LM, *P*=0.008, r^2^=0.72, df=91, F=25.8). (C) The increase in ^13/12^CO_2_ correlated to total Fe(II) concentrations (*P =*<0.0001). Fe(II)Cl_2_-treated sediments had a marginally higher ^13/12^CO_2_ compared to control sediment (LM, *P*=0.05, r^2^=0.76, df=90, F=30.1).

At the end of our 130-day incubation experiment the microbial community did not differ much from the in-situ community composition. This implies that the structure and composition of bacterial and archaeal communities was persistent and represented environmental conditions. Additionally, we found that the microbial community in both sediments had the potential to reduce Fe(III), as suggested by the 16S rRNA sequencing, and indicated by the increase in Fe(II) throughout the incubation experiment (Fig. 5A)_._ The Fe(II)-production rates were similar between H_2_O and ^13^CH_4_ treatment; which may be due to the active CH_4_ supply caused by the sediments’ high methanogenesis rates. Fe(III)-reducers, mainly affiliating with *Geobacteraceae* were present at similar abundances in both types of sediment (2.2% in the control and 2.6% in the FeCl_2_-treated enclosure), however, irrespective of the incubation treatment, in all the Fe(II)Cl_2_-treated sediments, the higher availability of Fe(III) minerals, resulted in a significantly higher concentration of total Fe(II) over time (LM, *P*=0.003, r^2^=0.80, df=91, F=37.7). However, part of the Fe(II) formation may also arise from anaerobic respiration of organic substrates. Additionally, we found that the headspace CH_4_ concentration correlated with total Fe(II) (*P=*<0.0001) concentrations, which is a proxy for Fe(III) reduction. Moreover, irrespective of the incubation treatment, total headspace CH_4_ concentration was lower in Fe(II)Cl_2_-treated sediments (Fig. 5B, LM, *P*=0.008, r^2^=0.72, df=91, F=25.8). This suggests that less CH_4_ is released when there is more Fe(III) reduction. This hypothesis is furthermore strengthened by the significant correlation between ^13/12^CO_2_ and total Fe(II) concentration, caused by Fe(III) reduction (LM, *P*=<0.0001). Fe(II)Cl_2_-treated sediments had a marginally higher ^13/12^CO_2_ compared to control sediments (Fig. 5C, LM, *P*=0.05, r^2^=0.76, df=90, F=30.1), as a result of more ^13^CH_4_ oxidation_._

In conclusion, we show that Fe(II)Cl_2_-treatment of eutrophic sediments in an urban pond led to significantly decreased aquatic CH_4_ emissions, which was likely facilitated by a rapid onset of Fe-AOM, without substantially changing the native microbial community. This highlights that Fe-AOM can be important in freshwater sediments. Therefore, in sediments where Fe(III) is the main alternative electron acceptor, Fe(II)Cl_2_ application has the potential to combat eutrophication as well as CH_4_ emissions, contributing to climate-smart water management.

## Supplementary Material

fiae061_Supplemental_File

## Data Availability

*All raw sequencing data have been deposited at the read sequence archive (SRA) database of the NCBI under the BioProject ID PRJNA966799 The remaining data will be uploaded in the repository, and will become available upon acceptance.
