# Supplementary material for: Fe(II)Cl2 amendment suppresses pond methane emissions by stimulating iron-dependent anaerobic oxidation of methane
Source: FEMS Microbiol Ecol. 2024 Apr 17;100(5):fiae061. doi: 10.1093/femsec/fiae061 (PMC11075768; doi:10.1093/femsec/fiae061)
Supplement: fiae061_Supplemental_File [file fiae061_supplemental_file.docx]

**TITLE:** *Fe(II)Cl_2_ amendment suppresses pond methane emissions by stimulating iron-dependent anaerobic oxidation of methane*

Quinten Struik^1,*^, José R. Paranaíba^1^, Martyna Glodowska^2^, Sarian Kosten^1^, Berber M. J. W. Meulepas^1^, Ana B. Rios-Miguel^2^, Mike S.M. Jetten^2^, Miquel Lurling^3^, Guido Waajen^4^, Thomas P.A. Nijman^1^, Annelies J. Veraart^1^

^1^ – Department of Ecology, Radboud Institute for Biological and Environmental Sciences, Radboud University, Nijmegen, The Netherlands

^2^ – Department of Microbiology, Radboud Institute for Biological and Environmental Sciences, Radboud University, Nijmegen, The Netherlands

^3^ – Aquatic Ecology & Water Quality Management Group, Department of Environmental Sciences, Wageningen University, P.O. Box 47, 6700 AA, Wageningen, The Netherlands

^4^ – Water Authority Brabantse Delta, Breda, The Netherlands

^*^Corresponding author – [Quinten.struik2@ru.nl](mailto:Quinten.struik2@ru.nl)

Radboud Institute for Biological and Environmental Science, Radboud University, Nijmegen, 6525AJ, The Netherlands

**Supplementary information**

This file contains the supplementary material for the paper: *Fe(II)Cl_2_ amendment suppresses pond methane emissions by stimulating iron-dependent anaerobic oxidation of methane*

**This document consists of:**

1 figure

2 tables

**Supplementary figures**


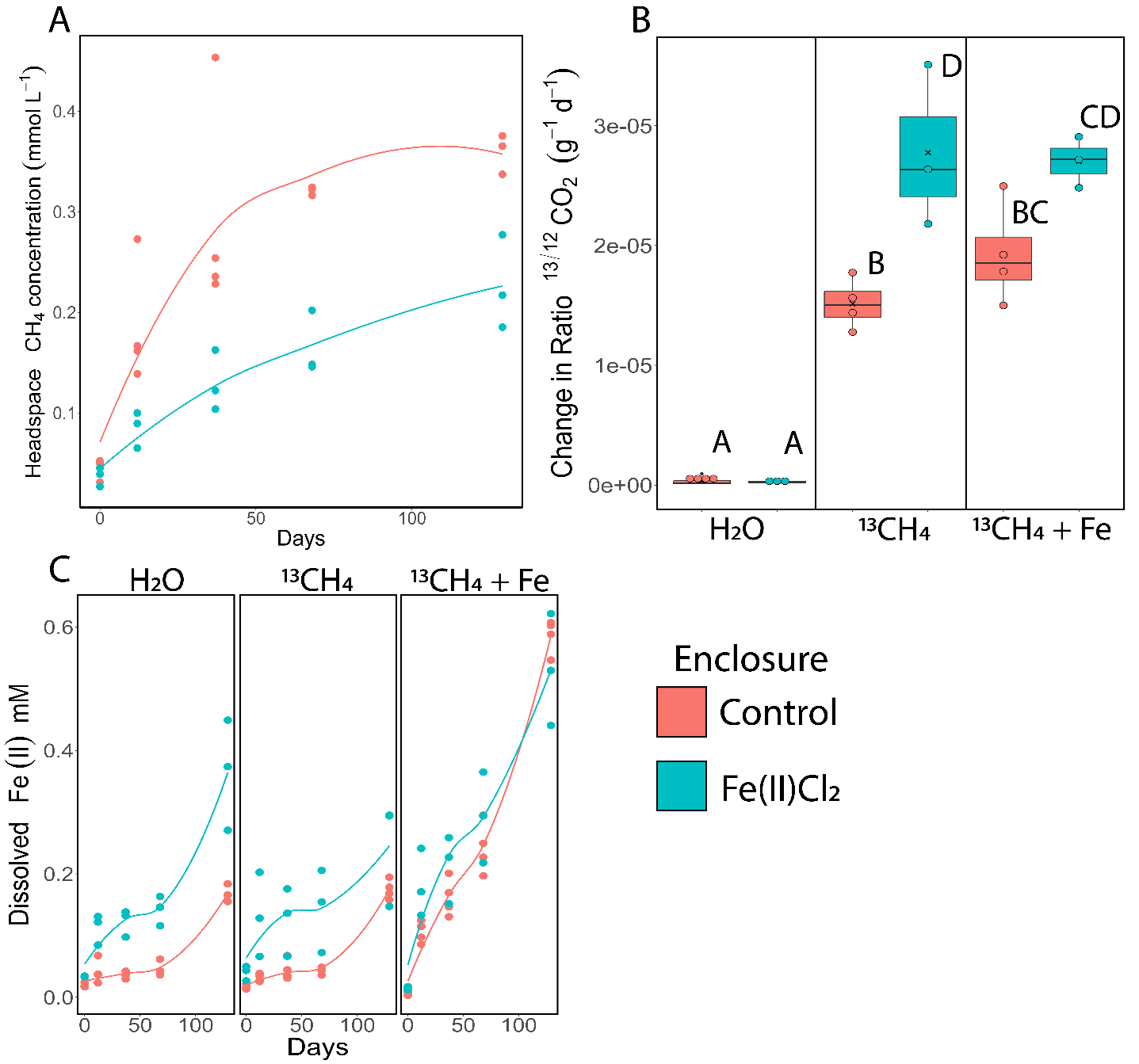


**Figure S1** **(a)** Net CH_4_-production, the development of CH_4_ concentration in the headspace throughout the batch incubations. **(b)** Change in ratio ^13/12^CO_2_ per calculated gram of dry sediment per day. The letters depict which groups significantly differ from each other based on a two-way ANOVA (*p=*<0.01, df=2, F=6.323) and Tukey post-hoc test (Table S1). **(c)** The change in dissolved Fe(II) concentration in mM throughout batch incubation. Boxes show the median and first and third quartiles, whiskers indicate upper and lower quartiles, dots indicate individual sampling points, and X indicates the mean

**Supplementary tables**

**Table S1:** Parameter estimates for **A)** the two-way ANOVA and for **B)** Tukey post-hoc test for the increase

in ratio ^13/12^CO_2_ during the batch incubation experiment.

| Source | Nparm | DF | Sum of squares | F-value | P-value |
| --- | --- | --- | --- | --- | --- |
| Treatment | 2 | 2 | 2.21e-9 | 99.81 | <.0001 |
| Enclosure | 1 | 1 | 2.36e-10 | 21.27 | 0.0003 |
| Treatment*Enclosure | 2 | 2 | 1.40e-10 | 6.32 | 0.0102 |
| Overall model | 5 | 5 | 2.49e-9 | 44.96 | <.0001 |

| Level (Enclosure; treatment) | -Level (Enclosure; treatment) | Mean difference | Std Err Difference | Lower CL | Upper CL | P-Value |
| --- | --- | --- | --- | --- | --- | --- |
| Fe(II)Cl_2_; ^13^CH_4_ | Fe(II)Cl_2_; H_2_O | 2.75E-05 | 2.72E-06 | 1.90E-05 | 3.63E-05 | <.0001 |
| Fe(II)Cl_2_; ^13^CH_4_ | Control; H_2_O | 2.74E-05 | 2.55E-06 | 1.90E-05 | 3.57E-05 | <.0001 |
| Fe(II)Cl_2_; ^13^CH_4_ + Fe | Fe(II)Cl_2_; H_2_O | 2.67E-05 | 2.72E-06 | 1.80E-05 | 3.56E-05 | <.0001 |
| Fe(II)Cl_2_; ^13^CH_4_ + Fe | Control; H_2_O | 2.67E-05 | 2.55E-06 | 1.80E-05 | 3.50E-05 | <.0001 |
| Control; ^13^CH_4_ + Fe | Fe(II)Cl_2_; H_2_O | 1.90E-05 | 2.55E-06 | 1.10E-05 | 2.72E-05 | <.0001 |
| Control; ^13^CH_4_ + Fe | Control; H_2_O | 1.89E-05 | 2.36E-06 | 1.10E-05 | 2.66E-05 | <.0001 |
| Control; ^13^CH_4_ | Fe(II)Cl_2_; H_2_O | 1.49E-05 | 2.55E-06 | 7.00E-06 | 2.31E-05 | 0.0004 |
| Control; ^13^CH_4_ | Control; H_2_O | 1.48E-05 | 2.36E-06 | 7.00E-06 | 2.25E-05 | 0.0002 |
| Fe(II)Cl_2_; ^13^CH_4_ | Control; ^13^CH_4_ | 1.26E-05 | 2.55E-06 | 4.00E-06 | 2.09E-05 | 0.0019 |
| Fe(II)Cl_2_; ^13^CH_4_ + Fe | Control; ^13^CH_4_ | 1.19E-05 | 2.55E-06 | 4.00E-06 | 2.02E-05 | 0.0033 |
| Fe(II)Cl_2_; ^13^CH_4_ | Control; ^13^CH_4_ + Fe | 8.50E-06 | 2.55E-06 | 0.00E+00 | 1.68E-05 | 0.0421 |
| Fe(II)Cl_2_; ^13^CH_4_ + Fe | Control; ^13^CH_4_ + Fe | 7.80E-06 | 2.55E-06 | 0.00E+00 | 1.60E-05 | 0.0714 |
| Control; ^13^CH_4_ + Fe | Control; ^13^CH_4_ | 4.10E-06 | 2.36E-06 | -4.00E-06 | 1.18E-05 | 0.5242 |
| Fe(II)Cl_2_; ^13^CH_4_ | Fe(II)Cl_2_; ^13^CH_4_ + Fe | 7.00E-07 | 2.72E-06 | -8.00E-06 | 9.60E-06 | 0.9998 |
| Control; H_2_O | Fe(II)Cl_2_; H_2_O | 1.00E-07 | 2.55E-06 | -8.00E-06 | 8.30E-06 | 1.0000 |

**A.
B.**

| Source | Nparm | DF | Sum of squares | F-value | P-value |
| --- | --- | --- | --- | --- | --- |
| Treatment | 2 | 2 | 0.37 | 28.67 | <.0001 |
| Enclosure | 1 | 1 | 0.08 | 11.66 | 0.0038 |
| Treatment*Enclosure | 2 | 2 | 0.08 | 6.25 | 0.0106 |
| Overall model | 5 | 5 | 0.58 | 18.14 | <.0001 |

**Table S2:** Parameter estimates for **A)** the two-way ANOVA and for **B)** Tukey post-hoc test for the dissolved Fe(II) concentration at the end of the batch incubation experiment.
**A.**

| Level (Enclosure; treatment) | -Level (Enclosure; treatment) | Mean difference | Std Err Difference | Lower CL | Upper CL | P-Value |
| --- | --- | --- | --- | --- | --- | --- |
| Control; ^13^CH_4_ + Fe | Control; H_2_O | 4.19E-01 | 5.66E-02 | 2.35E-01 | 6.03E-01 | <.0001 |
| Control; ^13^CH_4_ + Fe | Control; ^13^CH_4_ | 4.11E-01 | 5.66E-02 | 2.27E-01 | 5.95E-01 | <.0001 |
| Fe(II)Cl_2_; ^13^CH_4_ + Fe | Control; H_2_O | 3.63E-01 | 6.11E-02 | 1.65E-01 | 5.62E-01 | 0.0003 |
| Fe(II)Cl_2_; ^13^CH_4_ + Fe | Control; ^13^CH_4_ | 3.56E-01 | 6.11E-02 | 1.57E-01 | 5.54E-01 | 0.0004 |
| Fe(II)Cl_2_; ^13^CH_4_ | Control; H_2_O | 2.27E-01 | 6.11E-02 | 2.88E-02 | 4.26E-01 | 0.0206 |
| Control; ^13^CH_4_ + Fe | Fe(II)Cl_2_; H_2_O | 2.22E-01 | 6.11E-02 | 2.30E-02 | 4.20E-01 | 0.0247 |
| Fe(II)Cl_2_; ^13^CH_4_ | Control; ^13^CH_4_ | 2.20E-01 | 6.11E-02 | 2.13E-02 | 4.18E-01 | 0.0261 |
| Fe(II)Cl_2_; H_2_O | Control; H_2_O | 1.97E-01 | 6.11E-02 | -1.38E-03 | 3.96E-01 | 0.0521 |
| Control; ^13^CH_4_ + Fe | Fe(II)Cl_2_; ^13^CH_4_ | 1.91E-01 | 6.11E-02 | -7.25E-03 | 3.90E-01 | 0.0622 |
| Fe(II)Cl_2_; H_2_O | Control; ^13^CH_4_ | 1.90E-01 | 6.11E-02 | -8.95E-03 | 3.88E-01 | 0.0654 |
| Fe(II)Cl_2_; ^13^CH_4_ + Fe | Fe(II)Cl_2_; H_2_O | 1.66E-01 | 6.53E-02 | -4.63E-02 | 3.78E-01 | 0.1732 |
| Fe(II)Cl_2_; ^13^CH_4_ + Fe | Fe(II)Cl_2_; ^13^CH_4_ | 1.36E-01 | 6.53E-02 | -7.65E-02 | 3.48E-01 | 0.3476 |
| Control; ^13^CH_4_ + Fe | Fe(II)Cl_2_; ^13^CH_4_ + Fe | 5.55E-02 | 6.11E-02 | -1.43E-01 | 2.54E-01 | 0.9385 |
| Fe(II)Cl_2_; ^13^CH_4_ | Fe(II)Cl_2_; H_2_O | 3.02E-02 | 6.53E-02 | -1.82E-01 | 2.43E-01 | 0.9968 |
| Control; ^13^CH_4_ | Control; H_2_O | 7.57E-03 | 5.66E-02 | -1.76E-01 | 1.91E-01 | 1 |

**B.**
